# Using Big Data Analytics to “Back Engineer” Protein Conformational Selection Mechanisms

**DOI:** 10.3390/molecules27082509

**Published:** 2022-04-13

**Authors:** Shivangi Gupta, Jerome Baudry, Vineetha Menon

**Affiliations:** 1 Department of Computer Science, The University of Alabama in Huntsville, Huntsville, AL 35899, USA; sg0097@uah.edu; 2 Department of Biological Sciences, The University of Alabama in Huntsville, Huntsville, AL 35899, USA; jerome.baudry@uah.edu

**Keywords:** protein conformation selection, big data, deep learning, machine learning, feature selection, drug discovery

## Abstract

In the living cells, proteins bind small molecules (or “ligands”) through a “conformational selection” mechanism, where a subset of protein structures are capable of binding the small molecules well while most other protein structures are not capable of such binding. The present work uses machine learning approaches to identify, in a very large amount of protein:ligand complexes, what protein properties are associated with their capacity to bind small molecules. In order to do so, we calculate 40 physicochemical properties on about 1.5 millions of protein conformations: ligand and protein conformations. This work describes a machine learning approach to identify the unique physico-chemical descriptors of a protein that maximize the prediction rate of potential protein molecular conformations for the test case proteins ADORA2A (Adenosine A2a Receptor), ADRB2 (Adrenoceptor Beta 2) and OPRK1 (Opioid Receptor Kappa 1). We find adequate machine learning techniques can increase by an order of magnitude the identification of “binding protein conformations” in an otherwise very large ensemble of protein conformations, compared to random selection of protein conformations. This opens the door to the systematic identification of such “binding conformations” for proteins and provides a big data approach to the conformational selection mechanism.

## 1. Introduction

One of the frontier domains of biological research is to determine which small molecules, such as substrates or pharmaceuticals, are more likely than other small molecules to bind on a protein. Among the vast amount of small organic molecules present in living cells, only a small fraction—sometimes only a single chemical species—will normally bind to a specific protein. This specificity of protein:ligand is a central concept in chemical biology and in biochemistry, and the basis of virtually all projects in small molecules drug discovery. Yet, major questions remain on the mechanisms of protein:ligand specificity.

The widely accepted view of protein:ligand interactions is that a protein cycles through multiple conformations, and that a few of these protein conformations will be bound by the small molecules. Modern biophysical tools such as virtual docking [1], that predict the probability of a given small chemical to bind to a given protein, are used routinely in fundamental and industrial research, but they are limited because they usually consider only one protein conformation in an “induced fit” mechanism rather than an ensemble of conformations.

In our previous work we have performed massive systematic computational characterization of protein:ligands interactions for a very large number of protein conformations and small molecules. We have shown that we can identify the small number of protein conformations that will be “selected” for binding by their ligands. In principle these “binding” conformations will lead to free energy minima in the (protein + ligand) complex free energy hypersurface. In our research we are aiming to understand why these rare apo-conformations possess this capacity to bind their ligands, while the vast majority of the other protein conformations do not. Being able to successfully identify and predict the properties of binding conformations that render them able to bind their ligands would be a transformational change in our understanding of conformational selection, in that it will open the door to the prediction of which protein conformations should be used in the drug discovery pipeline that relies on computational docking. That will massively reduce the enormous cost of docking, by focusing on these binding confirmations alone. We also will answer several very important fundamental questions, beyond the nature of the protein properties responsible for conformational selection: are these properties similar for all proteins? Are they similar for classes of structurally close proteins? Or are they different from all proteins? Are these properties related also to the chemical properties of the ligands, as hypothesized behind most docking approaches? This work contributes to such characterization of what properties of an apo-protein conformation leads to conformational selection.

The fundamental approach is relatively simple: from our previous work we already [2] know which few protein conformations, among thousands, will lead to conformational selection, and we know which small molecules will be ligands of the proteins in these binding conformations. Calculating physico-chemical descriptors of the proteins, and building simple regression models could separate the protein conformations that bind ligands, from the protein conformations that do not bind ligands. However, the sheer amount of data renders “simple regression” calculations all but impossible. The data set we use here corresponds to about 1.5 millions of protein conformation and protein:ligand complex, and their associated interaction energies. A small fraction of this dataset contains a few hundreds of protein binding conformations that lead to conformational selection, and most of the rest of the data corresponds to protein conformations that are not statistically binding well to ligands. Calculating even “only” 40 physiochemical descriptors properties of the protein conformations, leads very quickly to a complexity that only Big Data Analytics can handle.

In addition, there are fundamental issues that are specific to the field of Big Data analytics that we aim at addressing in this work. Most of the real-world biomedical datasets suffer from statistical ill-conditioning issues such as class imbalance problem [3], which arises due to imbalanced groups or sub-categories present in the data. In this scenario, typically, the majority class or larger group of data that consists of non-binding protein conformations overshadows the minority class or smaller data group that comprises the data-of-interest, i.e., the binding protein conformations. In such a case, any machine learning (ML) technique applied for data-learning on a biased population dataset could result in higher misclassification of the smaller population of binding conformations, since the decision-making process is more biased towards the larger population of non-binding conformations. Conventional ML algorithms are not directly equipped to handle the class imbalance problem during the data-learning phase and therefore a new two-stage sampling-based classifier framework was proposed in our previous work [4,5] which aimed at tackling the class imbalance problem and maximizing the detection of potential binding conformations. This paper extends the use of the two-stage sampling-based classification approach with additional feature scoring and feature selection methods in conjunction with an Enrichment ratio framework, in order to validate and gain deeper understanding about the unique physico-chemical attributes of the selected protein conformation predictions from the ML framework. The feature scoring method uses three different feature selection methods to select the physio-chemical features or descriptors of potential drug-binding molecular conformations for target proteins ADORA2A, ADRB2 and OPRK1. The two-stage sampling-based classifier to maximize the prediction rate of potential binding conformations for target proteins ADORA2A, ADRB2 and OPRK1 and uses the proposed Enrichment ratio framework for the validation of the prediction results obtained from the ML framework. The goal of our ML-based feature analysis process is to advance our understanding of the significance of specific physico-chemical attributes or descriptors than make certain protein conformations more conducive to the protein:ligand binding process.

## 2. Background and Related Work

### 2.1. Analysis of Variance (ANOVA)

Analysis of Variance (ANOVA) is a statistical technique to calculate the linear dependency between each feature and the target variable and select the features with the highest F-values [6]. The ANOVA technique is performed between each feature and the target vector and the obtained F-value is assigned to that feature, wherein the features are ranked based on their information content. The higher the F-value, the more important is the feature. Here top ‘K’ features with the highest F-values were retained, where the K features to be selected is experimentally determined by the user. Therefore, ANOVA technique provides selection of primary physio-chemical protein features that play an important role in protein:ligand binding and conformation selection process.

### 2.2. Mutual Information (MI)

Mutual Information (MI) is defined as a measure between two random variables **X** and Y, that determines the amount of information obtained about one variable, through the other random variable [7]. In information theory, entropy is an important basis used for uncertainty or known information measurement. Given a variable **Y** and the conditional entropy **H**(**X**|**Y**) of **X** with respect to **Y** is defined as:

(1) $$H(X|Y)=-\sum_{y\in Y}^{\;}\sum_{x\in X}p\left(x,y\right)\;log(p(x|y))$$

(2) $$H(X)=-\sum_{x\in X}pp\left(x\right)\;log\left(p\left(x\right)\right)$$

where

- *p(x,y)* is the joint probability density function.
- *p(x|y)* is the posterior probabilities of **X** given **Y**.
- *p(x)* is the probability density function

From Equations (1) and (2), mutual information **I**(**X**;**Y**) [8] can be defined as below:

(3) $$I(X;Y)=H(X)-H(X|Y)=-\sum_{y\in Y}\sum_{x\in X}p\left(x,y\right)log\frac{p\left(x,y\right)}{p\left(x\right)\;p\left(y\right)}\;$$

Using Equation (3), if **X** and **Y** are independent of each other, i.e., if they are unrelated, then their MI value is 0. Similarly, if **X** and **Y** are dependent on each other, then their MI value is 1. Here independent implies that no information of **Y** can be obtained using **X** and dependent implies that we can determine **X** from **Y** or vice-versa. MI measure for the physio-chemical features can be determined as follows:

- Compute the MI to measure the dependency between the physio-chemical features vector and the target variable for all features.
- The features are then ranked based on their MI values.
- The top K features with highest MI values are retained where K is defined by the user.

Thus, MI explains the unique information present in the physio-chemical protein features that assists in protein conformation selection process.

### 2.3. Recurrence Quantification Analysis

Recurrence Quantification Analysis (RQA) is a numerical data analysis and statistical technique that is used for the study of non-linear dynamical systems [9]. The technique quantifies the number and duration of occurrences of a system presented by its state space trajectory. Recurrence analysis starts by quantifying the repeating patterns of the plot. Entropy of the distribution of the diagonal lines (***ENTR***), one of the measurements retrieved from the plots is the probability distribution *p*(*l*) of the diagonal line on the RQA plot is defined as:

(4) $$\mathrm{ENTR}=-\sum_{l=l_{min}}^{N}p\left(l\right)\;ln\left(p\left(l\right)\right)$$

where,

– *N* is the number of points on the state space trajectory
– *l* is the length of the diagonal line in the RQA plot

We evaluate the RQA-based entropy measure to understand the entropy with respect to time-space evolution of protein conformations and its relationship to probability of discovering potential binding conformation.

### 2.4. Logistic Regression

Logistic regression (LR) is a probabilistic statistical ML technique used to perform predictive analysis on data that is categorical or has binary classes. The main objective of LR is to determine the best fitting model that describes the relationship between a dependent binary variable against a group of independent variables [10]. Let *y* be the binary outcome indicating failure/success with values labeled as 0 or 1, respectively, and *p* be the probability of *y* = 1 as *p = 1*. Conversely, *y* = 0 can be expressed as 1 − *p*. LR then models the outcome *y* based on the linear combination of the independent data variables *b*_1_, *b*_2_, *…*, *b_n_* and their respective parameter/weight values A_1_, A_2_, …, A*_n_* via maximum likelihood method as given by:

(5) $$f\left(p\right)=log\frac{p}{p-1}={𝛽}_{0}+A_{1}b_{1}+A_{2}b_{2}+A_{3}b_{3}+\ldots +A_{n}b_{n}$$

LR technique is used in this work for supervised classification and prediction of potential binding vs. non-binding protein conformations selection.

### 2.5. SMOTE Algorithm

*Synthetic minority Over-sampling Technique* (SMOTE) is a well-liked method which helps in solving the class imbalance problem by creating new artificial data points rather than by over-sampling with replacement of the minority class [11]. The algorithm is elaborated below [12,13,14]:

- Select K neighbors.
- Oversampling of the minority class is carried out by taking the difference between the feature vector and its nearest neighbors.
- Multiply the difference obtained in the last step by a random weight between 0 and 1, and then add it to the feature vector under consideration. This causes the selection of an arbitrary point along the line segment between the two specific features.

This approach of the algorithm coerces the decision region of the minority class to be more general and therefore results in a desired well-balanced dataset.

### 2.6. Gaussian Naive Bayes Classifier

Gaussian Naive Bayes Classifier (GB) is a conditional probabilistic ML technique that is widely used for supervised data classification. According to Bayes theorem, the posterior probability can be expressed as the product of prior probability and likelihood as described by:

*P*(*y*|*x*) = (*P*(*x*|*y*) × *P*(*y*))/*P*(*y*) (6)

where,

- *P*(*y|x*) is called the posterior probability. It gives the probability of *y* given that the data point *x* is true.
- *P*(*x|y*) is called the likelihood. It gives the probability of point *x* given that the data point *y* is true.
- *P*(*y*) is called the prior probability of *y*. It gives the probability of *y* being true across all data points.
- *P*(*x*) is the probability of data point *x* averaged over all of the value of *y*.

Using Equation (6), posterior probability can be calculated for different features in the dataset. In case, when the features in the dataset has continuous values then the likelihood of the features is assumed to be Gaussian [15] and, hence, conditional probability is given by:

(7) $$P(x|y)\;\;\frac{1}{\sqrt{2\;\pi \;\sigma_{y}^{2}}}\exp\left(\frac{-{\left(x-\mu_{y}\right)}^{2}}{2\;\sigma_{y}^{2}}\right)$$

where,

- $\mu_{y}$ denotes the mean of class y
- $\sigma_{y}^{2}$ denotes the variance of class y

Since GB technique is probabilistic in nature, it is used in this work for supervised classification and prediction that is biased towards identification of potential binding protein conformations.

### 2.7. K-Nearest Neighbor Classifier

The K-Nearest Neighbors (KNN) algorithm is a non-parametric ML technique that is widely used for its easy interpretation and low calculation time for classification and regression problems. Given a testing example, the KNN algorithm directly searches through all the training examples by calculating the distances between the testing examples and the training dataset in order to identify its nearest neighbors and give a classification output [16]. The Euclidean distance, as defined in Equation (8), is used as the distance metric which assigns the testing example to the class among its k nearest neighbors (where k is an integer). KNN technique is another supervised classification method used in this work for prediction of potential binding vs. non-binding protein conformations selection.

(8) $$D\left(x,\;y_{i}\right)=\sqrt{{\left(x-y_{i}\right)}^{2}}$$

where,

- *x* is the new data pint
- $y_{i}$ is the data point in the training set

### 2.8. Confusion Matrix

In the ML supervised learning domain, confusion matrix is often used to represent the summary of classification or prediction results. The confusion matrix terminology for a binary classification has four cases that are used to interpret the classification/prediction results as follows:

- **True Positive (TP):** The case where the classifier correctly predicts ‘*binding*’ between the ligand and the target protein. (Class 1 right predictions)
- **True Negative (TN):** The case where the classifier correctly predicts ‘*No binding*’, i.e., the ligand and the target protein did not bind. (Class 0 right predictions)
- **False Negative (FN):** The case where the classifier predicts ‘*No binding*’, but the ligand and the target protein did bind. (Class 0 incorrect predictions)
- **False Positive (FP):** The case where the classifier predicts ‘*binding*’ but the ligand and the target protein did not bind. (Class 1 incorrect predictions)

Where class 0 refers to the non-binding conformations and class 1 denotes the potential binding conformations.

## 3. Proposed Methodologies

### ML-Based Feature Selection and Two Stage Sampling-Based Classification Framework

**Figure 1:**
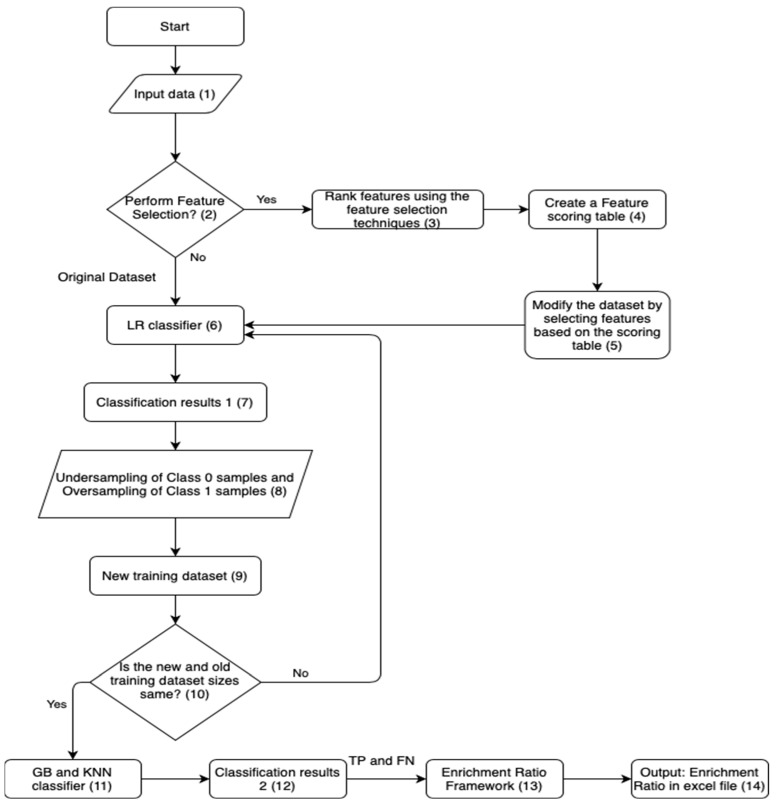
Flowchart of the proposed sampling-based framework with Enrichment ratio.

The proposed methodology aims to identify the recurrent unique physio-chemical protein features observed in the target proteins ADORA2A, ADRB2 and OPRK1 considered in our study. For this, we combine the feature selection techniques discussed in Section 2 with the two stage sampling-based classifier framework from our previous work [4,5] as outlined in steps below and illustrated in Figure 1:

- Input the dataset into the ML framework followed by the feature selection methods: (i) Analysis of variance (ANOVA), (ii) Mutual Information (MI) and (iii) Recurrence Quantification Analysis (RQA) are applied to the original dataset.
- The features identified as significant information pertinent to protein conformation binding are ranked based on F-value, MI score and Entropy using the ANOVA, MI and RQA methods, respectively.
- A feature scoring table is created based on the features ranks obtained from the three feature selection methods.
- A new dataset is then created based on the feature scoring table by selecting a common subset of features across the three feature selection methods.
- The LR classifier is applied to both the original dataset and the new modified dataset which is more biased towards class 0 samples. The identified class 0 (TN) and class 1 (TP) samples are recorded in the classification results 1.
- A new training dataset is created by applying the SMOTE algorithm to the original dataset and the new modified dataset.
- The SMOTE Algorithm performs random undersampling of class 0 samples based on the classification results of the LR classifier and oversampling of the desired class 1 samples. This step helps in tackling the class imbalance problem and also maximizes the detection rate of class 1 or active binding drug confirmation samples. For consistency, the size of the new training dataset is kept the same as the size of the original training dataset.
- After applying the SMOTE algorithm, the KNN and GB classifiers are applied to the new training dataset. The classification is performed to identify the binding conformations and non-binding conformations. The results from both the classifiers are recorded in classification results.
- Lastly, the True Positives (TP), i.e., binding conformations and the False negatives (FN), i.e., Inon-binding conformations obtained from the KNN and GB classification are used in calculating the Enrichment Ratios using the Enrichment ratio framework, in order to validate the ML prediction framework. The results from the Enrichment ratio framework are recorded.

## 4. Results and Discussion

### 4.1. Dataset Description

In our work, proteins ADORA2A (Adenosine A2a Receptor), ADRB2 (Adrenoceptor Beta 2) and OPRK1 (Opioid Receptor Kappa 1) were used for experimental validation of our proposed methods. The conformations of the three proteins have been thoroughly characterized, and the protein conformations that i) will bind to ligands (binding conformations) and ii) will not bind to ligands (non-binding conformations), are known and have been documented and published [2]. **ADORA2A**: The dataset has 50 attributes and consists of 2998 molecular conformations among which 851 molecular conformations are “binding” and 2147 molecular conformations that are “non-binding”.

**ADRB2**: The dataset has 51 attributes and consists of 2565 molecular conformations among which 156 are binding and 2411 molecular conformations are non-binding.

**OPRK1**: The dataset has 50 attributes and consists of 2998 molecular conformations among which 138 molecular conformations are binding and 2862 molecular conformations are non-binding. Table 1 below describes the protein descriptors for ADORA2A, ADRB2 and OPRK1 datasets that are used in this work.

### 4.2. Enrichment Ratios

In order to validate the ML protein conformation selection/prediction framework, the TP and FN predictions from the ML prediction framework described in Section 3 were used to calculate the enrichment ratio. A base enrichment ratio is calculated to gauge the generic prediction performance efficacy in the absence of the ML protein conformation selection framework. The base enrichment ratio was calculated by taking the number of binding conformations and dividing by the total number of conformations, from [2]. Equation (9) is used to calculate a baseline enrichment ratio that would have been found in testing stages if ML algorithms had not been implemented. Equation (10) was used to calculate an enrichment ratio from the predictions returned by the ML prediction framework. The values returned from both Equations (9) and (10) were then used to calculate the final enrichment ratios returned by each of the four filters as defined in Equation (11).

(9) $$\frac{number\;of\;known\;binding\;conformations}{total\;number\;of\;conformations\;\left(binding\;and\;non-binding\right)\;}\;=\mathrm{baseline}\;\mathrm{enrichment}\;\mathrm{ratio}$$

(10) $$\frac{number\;of\;binding\;conformations\;\left(TP\right)\;found\;in\;sample}{number\;of\;total\;conformations\;\left(TP\;and\;FN\right)\;found\;in\;sample}=\mathrm{ML}\;\mathrm{enrichment}\;\mathrm{ratio}$$

(11) $$\frac{ML\;enrichment\;ratio}{baseline\;enrichment\;ratio}\;=\mathrm{final}\;\mathrm{enrichment}\;\mathrm{ratio}$$

All “Filters” in the tables and text below refer to the compound selection methods described in Section 4.2.

### 4.3. Enrichment Ratio Framework

**Figure 2:**
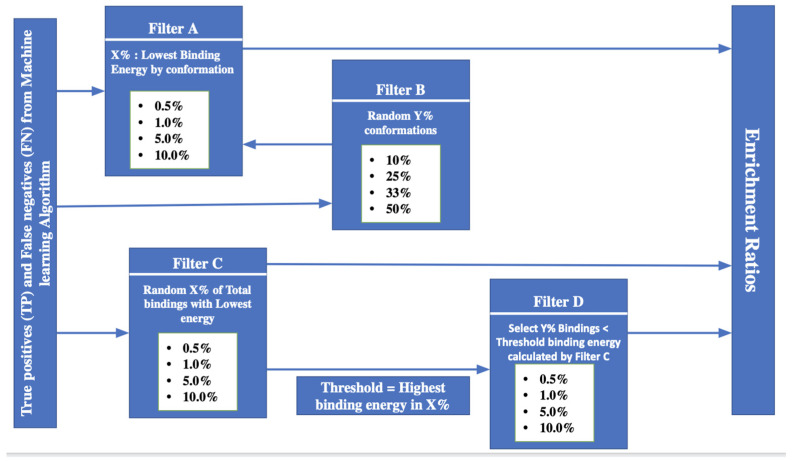
Enrichment ratio Framework.

The ML approaches discussed predict a binding/non-binding property for protein conformations without a specific “score” or a way to otherwise select rational subsets of the true positive (TP) and false negative (FN) predictions. However, in order to calculate the enrichment ratios as described in Equation (9) through (11), we have to select different subsets of the TP and FN results. In order to do this, we have chosen to base the subset data selection on the predicted protein:ligand interactions energies that were calculated and published previously [2]. The rationale is that, assuming that the calculated protein:ligand interaction energies are quantitatively correct, a “preferred” binding confirmation would be a conformations where the protein binds the ligand stronger (i.e., with better interaction energies), than in other conformations. Four different filters were used to calculate final enrichment ratios for proteins OPRK1, ADORA2A, and ADRB2 and are described below and are illustrated in Figure 2.

- **Filter A:** Takes all TP and FN conformations predicted by ML algorithms, and select a subset of a X% of the lowest energy for each individual conformation
- **Filter B:** Takes all TP and FN conformations predicted by ML algorithm and takes a random Y% of those conformations and returns back to filter A.
- **Filter C:** Takes all TP and FN conformations predicted by ML algorithm, sorts conformations by binding energy and takes a random X% of conformations with the lowest protein:ligand binding energy
- **Filter D:** Takes all TP and FN conformations predicted by ML algorithm and uses the maximum binding energy calculated from filter C and selects all binding energies inferior to that energy.

### 4.4. Computational Evaluation on ADORA2A Dataset

Table 2 gives the overview of Enrichment ratios that were calculated using the predicted binding conformations from the ML framework for ADORA2A. The ML framework was trained on 30% of the dataset and tested on the remaining 70% of the dataset, as shown in Table S1 through Table S3. It can be seen that the LR + SMOTE − KNN classifier gave the maximum enrichment ratio of 11.0, using data selection filter C (and about the same values when using LR + SMOTE − GB classifier data selection filter B).

### 4.5. Computational Evaluation on ADRB2 Dataset

Table 3 gives the overview of Enrichment ratios that were calculated using the predicted binding conformations from the ML framework for ADRB2. The ML framework was trained on 30% of the dataset and tested on the remaining 70% of the dataset. As in the ADORA2A case, the LR + SMOTE − KNN classifier gave the maximum enrichment ratio of 21.7, with data filter selection B.

### 4.6. Computational Evaluation on OPRK1 Dataset

Table 4 gives the overview of Enrichment ratios that were calculated using the predicted binding conformations from the ML framework for OPRK1. The ML framework was trained on 30% of the dataset and tested on the remaining 70% of the dataset. The LR + SMOTE − KNN classifier gave the maximum enrichment ratio of 20.1, using data filter selection A.

Table 5, Table 6 and Table 7 list out the physicochemical features common to different proteins. It can be observed from Table 5, that 5 features were common between ADORA2A and OPRK1. Table 6 lists the elements that were common between ADORA2A and ADRB2, it can be seen that only 1 feature is common between the two lists of Table 5 and Table 6. Table 7 lists the elements that are common between ADRB2 and OPRK1, it can be observed that 3 features are common between the two proteins. No features were found in common between the three proteins.

## 5. Conclusions

In this paper, we extended the use of the two-stage sampling-based classifier framework with additional feature scoring and feature selection methods in conjunction with Enrichment ratio framework, to identify the unique physico-chemical attributes of the binding conformation and based on those attributes understand what makes a protein conformation more conducive to *protein:ligand* binding process. The enrichments shown in Table 2 through Table 4 suggest that it is indeed possible to identify binding conformations in order of magnitude better than thought a random selection of protein conformations. This is a very encouraging result: it means that there are physico-chemical protein properties that will drive, at least in part, whether or not a protein conformation is more likely to be binding than non-binding. From a machine learning point of view, both LR + SMOTE − GB and LR + SMOTE − KNN classifiers demonstrated a reasonable prediction performance for binding protein conformations as described in (Table S1 through Table S3). In addition, the best enrichment is obtained for the smallest selection of binding conformations (0.5% to 1% of the conformation, see Table 2 through Table 4). This suggests that the “top” predicted binding conformations, in terms of strength protein: ligand energies as defined in Figure 2, contain the most “solid” data in terms of predictability power. This greatly simplifies the computational complexity and cost of this approach, as only a small fraction of the otherwise massive ensemble of conformations needs to be used in subsequent docking or analyzing.

There are however several challenges that limit, for the time being, the application of this approach to any protein conformation ensemble a priori. One limitation is that, as shown in Table 5 through Table 7, there does not seem to be a universal set of physico-chemical properties that are consistent across all the proteins studied here. Rather, it appears that each protein has its own unique set of “preferred” physico-chemical features that go into driving conformation selection. While this is not in itself a problem (indeed, individual proteins may very well have different physical and chemical ways to interact with their ligands), it certainly complicates the application of this approach to a novel protein for which no a priori knowledge of ligands does exist, and on which the model could be trained.

In our opinion, this suggests that there is a need to continue this work with (i) other proteins for which (preferably many) known ligands exist, to see how diverse are the physico chemical features in each set, and (ii) using different physicochemical descriptors that the 40 descriptors used here. The physico-chemical descriptors used here are well validated to quantify global protein properties and discriminate between global conformation, but it may be advantageous to use physic-chemical features that are limited to the binding sites of the proteins, where the interaction of the protein with its ligands are taking place. This is not however a trivial task. However, the fundamental result of this current work, that apo-protein conformations possess properties that correlate with conformational selection, makes this effort worthwhile.

Another challenge of this approach is a consequence of the severe class imbalance of the data, which contain much more conformations that are not selected by the ligands than conformations that are selected by the ligands. As shown in Tables S1–S3, the overall model accuracy for all the test case proteins is low due to the class imbalance problem in the dataset, but despite that the model was able to identify the potential binding protein conformations with a smaller training size which is depicted by the sensitivity score in the classification tables in Supplementary Materials. Table S1 for ADORA2A shows that the sensitivity score of LR + SMOTE − KNN method was 80% for a training size of only 30%. This indicates that the LR + SMOTE + KNN method was able to correctly predict 80% of binding protein conformations with only a 30% training size. Thus, the ML-based protein conformation framework described here performs well overall in detection of binding protein conformations, which is what the current work is focusing on. These results provide a baseline against which subsequent performance improvement benefits can be evaluated, in a generalized protein conformational selection model (enrichment ratio framework) through the adoption of ML-based prediction models.

The motivation of this work is to understand the protein properties responsible for conformation selection. Applied to novel target proteins, this approach yielded an enrichment ratio of ~11 (ADORA2A) to 20 (ADRB2, OPRK1). Using feature scoring, we observed 5 common physio-chemical descriptors between ADORA2A and OPRK1, 1 common descriptor between ADORA2A and ADRB2 and 3 common descriptors between ADRB2 and OPRK1. No common physio-chemical descriptors were found between ADORA2A, ADRB2 and OPRK1.

## Figures and Tables

**Table 1:** Describes the protein descriptors for ADORA2A, ADRB2 and OPRK1 datasets. The molecular descriptors were calculated using the *protein descriptors* of the program MOE [4,5,17].

Protein Property	Description
pro_pI_seq **	Sequence based pI
pro_mass	Protein Mass
pro_debye	Debye Screening length: Thickness of the Stern layer
pro_pI_3D	Structure-based pI Prediction
pro_coeff_280	Extinction coefficient at 280 nm
pro_coeff_fric	Frictional Coefficient
pro_coeff_diff	Diffusion coefficient
pro_r_gyr	Radius of Gyration
pro_r_solv	Hydrodynamic Radius
pro_sed_const	Sedimentation Constant
pro_eccen	Protein Eccentricity
pro_asa_vdw	Water Accessible Surface Area
pro_asa_hyd	Hydrophobic Surface Area
pro_asa_hph	Hydrophilic Surface Area
pro_volume	Protein Volume
pro_mobility	Protein Mobility
pro_helicity	Protein Helix Ratio
pro_henry	Henry’s Function f(ka)
pro_net_charge	Protein Net Charge
pro_app_charge	Protein Charge at Debye Length
pro_dipole_moment	Protein Dipole Moment
pro_hyd_moment	Hydrophobicity moment
pro_zeta	Zeta Potential
pro_zdipole	Zeta Dipole Moment
pro_zquadrupole	Zeta Quadrupole Moment
pro_patch_hyd	Area of hydrophobic protein patch(es)
pro_patch_hyd_1	Area of largest hydrophobic protein patch(es)
pro_patch_hyd_2	Area of 2 largest hydrophobic protein patch(es)
pro_patch_hyd_3	Area of 3 largest hydrophobic protein patch(es)
pro_patch_hyd_4	Area of 4 largest hydrophobic protein patch(es)
pro_patch_hyd_5	Area of 5 largest hydrophobic protein patch(es)
pro_patch_hyd_n	Count of hydrophobic protein patch(es)
pro_patch_ion	Area of ionic protein patch(es)
pro_patch_ion_1	Area of largest ionic protein patch(es)
pro_patch_ion_2	Area of 2 largest ionic protein patch(es)
pro_patch_ion_3	Area of 3 largest ionic protein patch(es)
pro_patch_ion_4	Area of 4 largest ionic protein patch(es)
pro_patch_ion_5	Area of 5 largest ionic protein patch(es)
pro_patch_ion_n	Count of ionic protein patch(es)
pro_patch_neg	Area of negative protein patch(es)
pro_patch_neg_1	Area of largest negative protein patch(es)
pro_patch_neg_2	Area of 2 largest negative protein patch(es)
pro_patch_neg_3	Area of 3 largest negative protein patch(es)
pro_patch_neg_4	Area of 4 largest negative protein patch(es)
pro_patch_neg_5	Area of 5 largest negative protein patch(es)
pro_patch_neg_n	Count of negative protein patch(es)
pro_patch_pos	Area of positive protein patch(es)
pro_patch_pos_1	Area of largest positive protein patch(es)
pro_patch_pos_2	Area of 2 largest positive protein patch(es)
pro_patch_pos_3	Area of 3 largest positive protein patch(es)
pro_patch_pos_4	Area of 4 largest positive protein patch(es)
pro_patch_pos_5	Area of 5 largest positive protein patch(es)
pro_patch_pos_n	Count of positive protein patch(es)

Note **: ADRB2 has 1 additional feature-pro_pl_seq.

**Table 2:** Enrichment Ratios of ADORA2A on the original dataset with no feature selection with training size of 30%.

Classifier	Maxima	Filter	% of Data Used	Minima	Filter	% of Data Used
LR + SMOTE − KNN	11.0	Filter C	0.5%	10.1	Filter A	0.5%
LR + SMOTE − GB	10.7	Filter B	0.5%	10.1	Filter C	1.0%

**Table 3:** Enrichment Ratios of ADRB2 on the original dataset with no feature selection with training size of 30%.

Classifier	Maxima	Filter	% of Data Used	Minima	Filter	% of Data Used
LR + SMOTE − KNN	21.7	Filter B	1.0%	11.2	Filter C	0.5%
LR + SMOTE − GB	8.3	Filter B	1.0%	4.2	Filter A	5.0%

**Table 4:** Enrichment Ratios of OPRK1 on the original dataset with no feature selection with a training size of 30%.

Classifier	Maxima	Filter	% of Data Used	Minima	Filter	% of Data Used
LR + SMOTE − KNN	20.1	Filter A	0.5%	18.5	Filter B	10%
LR + SMOTE − GB	13.3	Filter C	0.5%	3.9	Filter A	0.5%

**Table 5:** Common selected features between ADORA2A and OPRK1 having a feature score of 3.

pro_asa_vdw	pro_hyd_moment	pro_asa_hyd	pro_patch_neg_n	pro_zquadrapole

**Table 6:** Common selected features between ADORA2A and ADRB2 having a feature score of 3.

pro_hyd_moment

**Table 7:** Common selected features between ADRB2 and OPRK1 having a feature score of 3.

pro_patch_hyd	pro_patch_hyd_5	pro_patch_neg_1

## Data Availability

Statistical and computational models used are fully detailed in the main text. Data will be made available upon personal requests to authors.

## Supplementary Materials

The following are available online at https://www.mdpi.com/article/10.3390/molecules27082509/s1, Table S1: Classification table of ADORA2A with a training size of 30%, Table S2: Classification table of ADRB2 with a training size of 30%, Table S3: Classification table of OPRK1 with a training size of 30%.

## Sample Availability

Samples of the compounds are not available from the authors.
