# Supplementary material for: Using Big Data Analytics to “Back Engineer” Protein Conformational Selection Mechanisms
Source: Molecules. 2022 Apr 13;27(8):2509. doi: 10.3390/molecules27082509 (PMC9025728; doi:10.3390/molecules27082509)
Supplement: Supplementary file 1 [file molecules-27-02509-s001.zip › molecules-1601313-SI.pdf]

molecules MDPI

Article

Using Big Data Analytics to "Back Engineer" Protein Conformational Selection Mechanisms.

Shivangi Gupta [1], Jerome Baudry [2], and Vineetha Menon [1,*] [1]

Table S1. Classification table of ADORA2A with a training size of 30%

Class 0 (number of non-binding conformations) samples		Class 1 (number of binding conformations) samples		Classifier	TP	FN	Accuracy (%)	Sensitivity(%)
Training	*Testing*	*Training*	*Testing*					
652	1498	248	602	LR+SMOTE-GB	345	257	56.0	57.0
				LR+SMOTE-KNN	479	123	47.0	80.0

Table S2. Classification table of ADRB2 with a training size of 30%

Class 0 (number of non-binding conformations) samples		Class 1 (number of binding conformations) samples		Classifier	TP	FN	Accuracy (%)	Sensitivity (%)
Training	*Testing*	*Training*	*Testing*					
719	1692	50	104	LR+SMOTE-GB	21	83	82.5	20.0
				LR+SMOTE-KNN	34	70	67.1	33.0

Table S3. Classification table of OPRK1 with a training size of 30%

Class 0 (number of non-binding conformations) samples		Class 1(number of binding conformations) samples		Classifier	TP	FN	Accuracy (%)	Sensitivity (%)
Training	*Testing*	*Training*	*Testing*					

858	2004	41	96	LR+SMOTE-GB	54	42	57.6	56.0
				LR+SMOTE-KNN	47	49	57.6	49.0
